# Social engagement and loneliness are differentially associated with neuro-immune markers in older age: Time-varying associations from the English Longitudinal Study of Ageing

**DOI:** 10.1016/j.bbi.2019.08.189

**Published:** 2019-11

**Authors:** Emma Walker, George Ploubidis, Daisy Fancourt

**Affiliations:** a Department of Epidemiology and Public Health, University College London, United Kingdom; b UCL Centre for Longitudinal Studies, Department of Social Science, University College London, United Kingdom; c Department of Behavioural Science and Health, University College London, United Kingdom

**Keywords:** Inflammation, Loneliness, Social isolation, Social support, CRP, Fibrinogen, IGF-1

## Abstract

**Objectives:**

This study aimed to explore time-varying associations between social engagement, living status and loneliness and neuro-immune markers in older adults, and ascertain whether results are explained by socioeconomic position, health behaviours or depression.

**Methods:**

We analysed blood samples from 8780 adults aged 50 and above from the English Longitudinal Study of Ageing across three waves of data collection: 2004/5, 2008/9 and 2012/2013. We used fixed effects modelling to estimate the relationship between loneliness, social isolation, living alone and levels of fibrinogen, insulin like growth factor-1 (IGF-1), white blood cell (WBC) count and C-reactive protein (CRP), whilst accounting for all time-invariant and identified time-varying confounders.

**Results:**

Higher levels of social engagement and living with somebody were associated with lower levels of CRP, fibrinogen and WBC, while lower levels of loneliness were associated with higher levels of IGF-1. These associations were found to be independent of time-invariant factors such as gender, medical history, previous patterns of social behaviours, unobserved aspects of social class, and genetics, and time-varying factors such as income, physical health, health behaviours, and depression.

**Conclusions:**

Aspects of social engagement were associated with lower levels of inflammation whilst loneliness was inversely related to the regulation of inflammation. This suggests there could be different biological pathways involved in objective and subjective aspects of social connections.

## Highlights

• Social engagement & cohabitation are associated with lower CRP, fibrinogen & WBC.
• Lower levels of loneliness are longitudinally associated with higher levels of IGF-1.
• Associations are independent of time-constant factors e.g. gender, social class.
• Associations are not explained by time-varying factors e.g. income, or depression.
• Isolation is related to inflammation & loneliness to the regulation of inflammation.

## 1 Introduction

Multiple aspects of social connection, including social engagement (an individual’s *quantity* of social contact) and loneliness (an individual’s *quality* of social interactions) have been linked to both morbidity and mortality (Steptoe et al., 2013b). A number of psychological and biological pathways have been identified to explain this relationship (Cacioppo et al., 2015, Cacioppo and Hawkley, 2009), including a bidirectional regulatory role of inflammatory processes (Eisenberger et al., 2017). Social stressors such as social disengagement can lead to upregulation of pro-inflammatory response genes to protect against physical vulnerability (Cole et al., 2007), and this may be detected as higher levels of a range of inflammatory markers including interleukin-6 (IL-6), interleukin-1 receptor alpha (IL-1Ra), fibrinogen and C-reactive protein (CRP) (Hackett et al., 2012). These inflammatory responses are bidirectionally interlinked (Leng et al., 2005, Wong et al., 2007), so the analysis of multiple biomarkers related to inflammation together can provide an overall indication of inflammatory response. However, individual biomarkers can also play different functional roles within the immune system and can act as independent predictors of health outcomes (The Emerging Risk Factors Collaboration, 2012, Willems et al., 2010). Therefore, studies of social stressors have frequently considered multiple inflammatory markers simultaneously.

Exploring the reciprocal pathway, increases in inflammation have also been linked with social outcomes such as social anhedonia (Hannestad et al., 2011), social disconnection (Moieni et al., 2015), and loneliness (Eisenberger et al., 2010). Pro-inflammatory states can increase sensitivity to negative social experience such as rejection (Eisenberger et al., 2003), although some studies have suggested that approach-related behaviour, such as seeking help or care, can also be increased (Inagaki et al., 2015, Moieni and Eisenberger, 2018). Furthermore, inflammatory activity is bi-directionally associated with chronic stress and depression (Reichenberg et al., 2001), which themselves have been linked with both loneliness and social disengagement, and less effective regulation of pro-inflammatory activity (Avitsur et al., 2001, Dowlati et al., 2010).

However, it remains unclear if there are differential biological responses to social disengagement versus loneliness. Only a few, mainly cross-sectional, studies have made direct and simultaneous comparisons (Shankar et al., 2011). But such investigations are restricted by a high possibility of reverse causation. In addition, they cannot consider how changes in social engagement over time may relate to changes in biomarkers, nor the role that changes in individual life experiences and both physical and mental health might play in confounding associations. This is especially important when considering the elderly population as loneliness and social disengagement can result from life events such as the loss of a partner or the onset of a disability (Dykstra et al., 2005), and have complex and dynamic associations with low mood, physical activity and a general decline in activity in older age (Singh and Misra, 2009). Therefore, this study assessed time-varying associations between social engagement, living status, loneliness and neuro-immune markers in older adults and explored the potential confounding roles of factors including socioeconomic position (SEP), sedentary behaviours and depression.

Methods

### 1.1 Participants

We analysed data from the English Longitudinal Study of Ageing (ELSA): a nationally-representative longitudinal cohort study of adults over the age of 50 living in England (Steptoe et al., 2013a). The sample was drawn from households that had participated in Health Survey for England (HSE) in 1998, 1999, and 2001 (wave 0). HSE uses multi-stage stratified probability sampling with postcode sectors selected at the first stage and household addresses selected at the second stage to recruit participants. For the present analyses, we used data collected in waves 2 (2004/2005), 4 (2008/2009) and 6 (2012/2013) of ELSA; the three waves in which blood samples were taken during nurse visits. 8780 core participants provided data in wave 2 and we used multiple imputation for missing values to maintain the sample size across the two waves of follow-up. The data are available through the UK Data Service and ethical approval for ELSA was provided by the National Research Ethics Service. All research was performed in accordance with research and data protection guidelines with all respondents providing informed consent.

### 1.2 Measures

*Social Engagement* was measured using self-report scales that assessed the frequency of social interactions. This included face to face interaction with children, other family members or friends, participation in community group activities (including political party, trade union or environmental groups, tenant groups, resident groups, neighbourhood watch groups, church or other religious groups, charitable associations, education, arts or music groups or evening classes, social clubs, sports clubs, exercise classes, or any other organisations, clubs or societies), and engagement with community cultural activities (including going to museums, exhibitions, the theatre, concerts, opera or the cinema). The frequency of face-to-face interaction was measured as less than once a month, once or twice a month, once or twice a week, or three or more times a week. The frequency of community group participation was measured as never, once or twice a year, every few months, or monthly or more. The frequency of cultural engagement was measured as never, less than once a year, once or twice a year, or every few months or more. All of these four-point scales were scored from 1 to 4 and these scores were then summed to provide an overall participation index of 3 to 12, with higher scores indicating higher levels of social engagement. Factor analysis using Kaiser’s criterion of eigenvalues >1 confirmed that the items were a single factor (with Kaiser-Meyer-Olkin confirming sampling adequacy = 0.71), and the scale had acceptable internal consistency (Cronbach’s alpha = 0.65).

*Living status* was measured through self-report of the number of individuals living in the household during participant interviews and was collapsed to a binary variable of living alone vs living with one or more people.

*Loneliness* was measured using an adapted 3-item questionnaire (Hughes et al., 2004) based on the UCLA loneliness scale (Russell et al., 1978). ELSA respondents were asked how often they felt they (i) lacked companionship (ii) felt left out and (iii) felt isolated from others around them. Frequencies were hardly ever or never (assigned a score of 3), some of the time (assigned a score of 2), and often (assigned a score of 1). The scores for each measure were then summed to give a loneliness score ranging from 3 to 9 where lower scores indicated higher levels of loneliness. The scale had good internal consistency in line with validations (Cronbach’s alpha = 0.77).

*Neuro-immune Biomarkers.* Blood samples collected during ELSA nurse visits were analysed to give data on a range of biomarkers. Of these, the blood concentration of four neuro-immune markers were measured, and all four of these were therefore used in these analyses: fibrinogen (g/L), insulin like growth factor-1 (IGF-1; mmol/L), white blood cell count (WBC; analysed as continuous counts per 10^9^/L), and C-reactive protein (CRP; mg/L). Fibrinogen was measured using a modification of the Clauss thrombin clotting method on the Organon Teknika MDA 180 analyser. IGF-1 was measured using the DPC Immulite 2000 method. CRP was measured using the N Latex CRP mono immunoassay on the Behring Nephelometer II analyser. All blood samples were analysed at the Royal Victoria Infirmary laboratory in Newcastle upon Tyne, UK (for a detailed description of blood analyses see Graig et al., 2004). WBC and IGF-1 levels were reported at waves 4 and 6 only, therefore multiple imputation was additionally used to provide values for WBC and IGF-1 at wave 2 (see below). For CRP levels, we excluded participants with results of higher than 10 mg/L, since these may indicate the presence of an acute infection or serious acute illness, and results were log-transformed to ensure a normal distribution. Other biomarkers showed a normal distribution.

*Covariates.* A series of time-varying covariates were considered: respondent marital status (single/widowed/divorced vs married/cohabiting); employment status (working part/full time vs not working); total non-pension wealth in quintiles (Banks et al., 2010); presence of a long standing illnesses (diagnosis of cancer, COPD, arthritis, stroke, diabetes and angina or depression); long term moderate or severe chronic pain; frequency of alcohol consumption (less than once a week, 1–2 times a week, 3–4 times a week or 5+ times a week); current smoking status; sedentary behaviours (engaging in mild, moderate or vigorous sports or activities less than once a week); depression (using a score of 3+ on the Centre for Epidemiological Studies Depression scale CES-D, which is used as an alternative to 4+ to include broader depressive symptoms that could be associated with differences in biological markers) (Turvey et al., 1999, White et al., 2016); and body mass index (BMI).

### 1.3 Analysis

Analyses were carried out using Stata 14 (StataCorp, College Station, TX). 26.8% of total data items were missing and assumed to be missing at random so were imputed using multiple imputation by chained equations using the following predictor variables (in addition to variables in the substantive model): economic factors (employment status, wealth); social indicators (cultural engagement, number of friends, social engagement, positive and negative social interactions, whether respondents had a hobby); health behaviours (alcohol consumption, smoking status, sedentary behaviours); objective and subjective health measures (chronic health conditions, reported long term pain, self-rated health); daily newspaper reading and nurse visit data (waist hip ratio, body mass index, HDL to total cholesterol ratio, glycated haemoglobin blood concentration, resting pulse, systolic and diastolic blood pressure, triglyceride blood concentration and waist circumference). We undertook a single set of imputations for all missing data together and 50 imputations were conducted.

Fixed-effects regression was used to estimate the relationship between loneliness, social engagement, living with somebody and levels of the four biomarkers. This approach has several strengths. First, fixed-effects regression considers time-varying relationships. This is particularly helpful when exploring social factors such as loneliness and social engagement as these are likely to change as people age and are also likely to be influenced by other time-varying factors such as health or retirement status. Second, in fixed-effects regression, within-person variation is explored with individuals acting as their own reference point over time. Therefore all time-invariant factors (e.g. sex, ethnicity, genetics, personality, educational attainment and socio-economic position) are accounted for even if unobserved (Allison, 2009) and so do not need to be included in statistical models (and are shown in Table 1 purely for descriptive purposes).

**Table 1:** Participant characteristics at baseline.

Time-invariant characteristics (stated at baseline) a		Proportion (%)
Gender	% female	55.0%
Age	% 50–55	5.3%
	% 56–60	15.2%
	% 61–65	20.3%
	% 66–70	17.4%
	% 71–75	15.3%
	% 76+	26.4%
Ethnicity	White, %	97.7%
Educational Attainment^b^	No qualifications / basic qualifications	44.2%
	GCSE / O-level / qualification at age 16	16.7%
	A-levels / higher education / qualification at age 18	27.1%
	Degree / further higher qualification	12.1%

Time-varying characteristics (state at baseline)		
Marital Status	Married/cohabiting, %	66.4%
Employment	Working full- or part-time, %	30.9%
Wealth	Measured in quintiles	–
Alcohol Consumption	Less than once a week	48.9%
	Once or twice a week	20.9%
	3 or 4 times a week	10.6%
	5 or more times a week	19.7%
Smoking Status	Smoker, %	11.3%
Sedentary Behaviour	Exercises less than weekly, %	8.7%
Chronic Health Conditions	One or more of cancer, COPD, arthritis, stroke, diabetes, angina, %	35.1%
Chronic pain	Experiencing moderate or severe chronic pain, %	22.3%
Depression	Score of 3+ in CES-D, %	17.1%
BMI	Mean score	27.6 (SE 0.04)

Social connections		
Social engagement	Mean score (range 3–12; higher indicates more socially engaged)	7.9 (SE 0.02)
Living with somebody	%	78.0%
Loneliness	Mean score (range 3–9; lower indicates more lonely)	7.7 (SE 0.01)

a Excluded from the analysis as time-invariant factors are automatically included within fixed-effects models, but shown here for descriptive purposes.

The basic model for the analysis can be expressed as follows:

$${\mathrm{Bio}}_{\mathrm{it}}={\beta_{0t}+\beta_{1}S}_{\mathrm{it}}+\beta_{2}T_{\mathrm{it}}+\alpha_{i}+\varepsilon_{\mathrm{it}}$$

where Bio_it_ is a measure of individual i's levels of neuro-immune markers at time t, α_i_ is unobserved time invariant confounding factors, S is whether an individual was experiencing loneliness, social engagement or living alone at time t, and T is measured time-varying confounding factors. Data were strongly balanced. A Hausman test was used to confirm the selection of a fixed effects over a random effects model. The modified Wald test for group-wise heteroscedasticity was significant, so sandwich estimators were applied. Coefficients for all years were not jointly equal to zero, so time-fixed effects were included in the model.

Model 1 was unadjusted for time-varying factors, but automatically adjusted for all time-invariant factors. Model 2 additionally adjusted for time-varying factors demographic factors (marital status, employment status and wealth), model 3 additionally adjusted for time-varying health-related factors (chronic illness, chronic pain, alcohol consumption, smoking and sedentary behaviours), and model 4 additionally adjusted for depression. A sensitivity analysis additionally adjusted for BMI, given known covariation with biological markers. A further sensitivity analyses explored whether there was evidence of a moderating role of gender by including interaction terms in the analyses. Additionally, we excluded participants who had experienced an acute infection in the three weeks prior to blood sampling at any of the three time points. We also applied propensity weights to our analyses to ensure the sample was representative. Moreover, as we had entirely imputed data for WBC & IGF-1 at wave 2, we additionally ran analyses not using this imputed wave.

Amongst our sample, objective and subjective markers of social engagement were only associated to a small degree (lower levels of loneliness and social engagement: r = 0.21, p < .001; lower levels of loneliness and living with somebody r = 0.30, p < .001). Living with somebody and social engagement were also only minimally associated (r = 0.08, p < .001). Therefore, each model included all three social exposures simultaneously in order that they mutually adjusted for one another, but a further sensitivity analysis additionally examined each social predictor in a model of its own.

## 2 Results

### 2.1 Participants

Of the 8780 participants, 55% were female with age range 52–99. The majority were married or in a partnership (66.4%) and had no or basic qualifications only (44.2%). At baseline, just under a third of the sample (30.9%) were employed in either full time or part time work. Full details of the sample are provided in Table 1.

### 2.2 Social engagement

Higher levels of social engagement were associated with lower levels of CRP, fibrinogen and WBC count in minimally-adjusted models (Table 2). However, in the fully adjusted model, only the associations for fibrinogen (coef −0.012, 95% CI −0.021 to −0.003) and WBC count (coef −0.040, 95% CI −0.078 to −0.002) remained. Social engagement was not associated with IGF-1 in any model.

**Table 2:** Results from fixed-effects regression models showing time-varying associations between loneliness, engagement, living alone and neuro-immune markers.

	CRP (95% CI)		Fibrinogen (95% CI)		WBC (95% CI)		IGF-1 (95% CI)	
	Coef (95% CI)	p	Coef (95% CI)	p	Coef (95% CI)	p	Coef (95% CI)	p
*Model 1: accounting for all time-invariant factors*								
Social engagement	**−0.018 (−0.026 to −0.010)**	**<0.001**	**−0.025 (−0.034 to −0.015)**	**<0.001**	**−0.077 (−0.114 to −0.040)**	**<0.001**	0.012 (**−**0.084 to 0.108)	0.80
Living with somebody	**−0.167 (−0.208 to −0.126)**	**<0.001**	**−0.219 (−0.269 to −0.015)**	**<0.001**	**−0.539 (−0.713 to −0.365)**	**<0.001**	**0.753 (0.272 to 1.232)**	**0.002**
Low levels of loneliness	**−0.019 (−0.030 to −0.007)**	**0.002**	**−0.017 (−0.030 to −0.004)**	**0.008**	**−0.061 (−0.107 to −0.015)**	**0.010**	**0.184 (0.082 to 0.286)**	**<0.001**

***Model 2: additionally adjusted for time-varying demographic factors***								
Social engagement	**−0.010 (−0.019 to −0.002)**	**0.014**	**−0.017 (−0.026 to −0.008)**	**<0.001**	**−0.060 (−0.098 to −0.022)**	**0.003**	−0.024 (**−**0.122 to 0.073)	0.62
Living with somebody	**−0.086 (−0.126 to −0.046)**	**<0.001**	**−0.131 (−0.181 to −0.081)**	**<0.001**	**−0.352 (−0.541 to −0.183)**	**<0.001**	0.356 (**−**0.112 to −0.824)	0.14
Low levels of loneliness	−0.010 (**−**0.021 to 0.002)	0.10	−0.007 (**−**0.020 to 0.005)	0.24	−0.040 (**−**0.086 to 0.006)	0.089	**0.139 (0.037 to 0.241)**	**0.008**

***Model 3: additionally for time-varying health-related factors***								
Social engagement	−0.007 (**−**0.015 to 0.001)	0.098	**−0.012 (−0.021 to −0.003)**	**0.007**	**−0.040 (−0.078 to −0.003)**	**0.037**	−0.026 (**−**0.124 to 0.071)	0.59
Living with somebody	**−0.059 (−0.099 to −0.019)**	**0.004**	**−0.100 (−0.150 to −0.051)**	**<0.001**	**−0.249 (−0.424 to −0.074)**	**0.006**	0.313 (**−**0.155 to 0.781)	0.19
Low levels of loneliness	−0.005 (**−**0.016 to 0.007)	0.43	−0.002 (**−**0.014 to −0.010)	0.76	−0.020 (**−**0.065 to 0.026)	0.39	**0.132 (0.029 to 0.235)**	**0.012**

***Model 4: additionally for time-varying depression***								
Social engagement	−0.007 (**−**0.015 to 0.001)	0.11	**−0.012 (−0.021 to −0.003)**	**0.008**	**−0.040 (−0.078 to −0.002)**	**0.041**	−0.026 (**−**0.124 to 0.072)	0.60
Living with somebody	**−0.057 (−0.097 to −0.018)**	**0.004**	**−0.098 (−0.147 to −0.048)**	**<0.001**	**−0.238 (−0.416 to −0.060)**	**0.009**	0.315 (**−**0.151 to 0.781)	0.18
Low levels of loneliness	−0.004 (**−**0.015 to 0.008)	0.55	−0.001 (**−**0.013 to 0.012)	0.91	−0.014 (**−**0.060 to 0.032)	0.54	**0.133 (0.026 to 0.240)**	**0.015**

Number of observations: 26,340; number of individuals: 8780; observations per group: 3. **Higher scores indicate greater social engagement, living with others and lower levels of loneliness.** Model 1 accounted for all time-invariant factors, even if unobserved. Model 2 adjusted for time-varying demographic covariates (marital status, employment status, wealth). Model 3 additionally adjusted for time-varying health-related factors (presence of a long standing illnesses, long term pain, alcohol consumption, smoking status, sedentary behaviours). Model 4 additionally adjusted for depression.

### 2.3 Living with somebody

Living with somebody was associated with lower levels of CRP, fibrinogen and WBC count, even in fully-adjusted models (CRP: coef −0.057, 95% CI −0.097 to −0.018; fibrinogen: coef −0.098, 95% CI −0.147 to −0.048; WBC count: coef −0.238, 95% CI −0.416 to −0.060) (Table 2). Living with somebody was only associated with higher levels of IGF-1 when just time-invariant factors were controlled for and results were attenuated when considering time-varying demographic factors.

### 2.4 Loneliness

Lower levels of loneliness were associated with lower levels of CRP, fibrinogen and WBC count when accounting just for time-invariant factors (Table 2). However, these associations were attenuated when considering time-varying demographic factors. Conversely, low levels of loneliness were associated with higher levels of IGF-1, independently of all time-invariant and identified time-varying factors (coef 0.133, 95% CI 0.026 to 0.240).

### 2.5 Sensitivity analyses

When the fully adjusted models were additionally controlled for BMI, all results were maintained (see Supplementary Table 1). Independent analysis of each exposure made only slight changes to the regression coefficients observed but no material changes to the findings (see Supplementary Table 2). Results were also maintained when excluding individuals who had experienced an infection and when weighting using survey weights (Supplementary Tables 3 & 4). There was no evidence of any moderating role of gender for any of the exposures or outcomes. When analysing data for WBC and IGF-1 not including the imputed data for wave 2, although the pattern of findings were maintained, significance was lost in the more adjusted models (see Supplementary Table 5).

## 3 Discussion

This study was the first to simultaneously examine social engagement, living with somebody, loneliness and biomarkers in longitudinal data and showed differential associations with CRP, fibrinogen, WBC count and IGF-1. Higher levels of social engagement and living with somebody were associated with lower levels of CRP, fibrinogen and WBC, while lower levels of loneliness were associated with higher levels of IGF-1. These associations were found to be independent of time-invariant factors such as gender, medical history, previous patterns of social behaviours, unobserved aspects of social class, and genetics, and independent of time-varying factors such as income, physical health, and health behaviours. Notably, despite the strong literature linking depression with inflammation, the associations were also found to be independent of depression. This echoes a range of previous studies that found associations between aspects of social connection and inflammation independently of depression, and suggests that depression does not entirely explain the biological impact of loneliness and social disengagement (Cankaya et al., 2009, Cole et al., 2015, Kim et al., 2016).

Previous studies have suggested a link between social connectedness and living with a spouse and lower fibrinogen (Kim et al., 2016, Ploubidis et al., 2015), diminished regulation of WBC trafficking (Cole, 2008, Ploubidis et al., 2015), and lower CRP (Ford et al., 2006, Heffner et al., 2011, Sbarra, 2009). However, although our study confirmed the finding for living with somebody, it also showed that the association between social engagement and CRP is attenuated when considering health-related factors not included in previous studies such as physical illness. One previous study did report the same result as in this study for CRP in men but not women (Ford et al., 2006), but we found no evidence of a moderating role of gender across any of the biomarkers.

With respect to loneliness, we did not find associations with CRP, fibrinogen or WBC. This goes against some previous studies (Mezuk et al., 2016, Nersesian et al., 2018). However, it is of note that some of these studies have been cross-sectional. Further, our findings for CRP and fibrinogen are supported by some other studies (Shankar et al., 2011, Yang et al., 2013). We did, however, find an association between loneliness and IGF-1. Increases in IGF-1 have previously been linked with the death of close friends or partners (Cankaya et al., 2009). The authors had suggested trauma as a mediator of the association. However, given our findings it is possible that incurred loneliness could be another explanatory factor. IGF-1 regulates cell growth, and has been implemented in the ageing process (Junnila et al., 2013) but also in psychosocial outcomes such as depression (Chigogora et al., 2016). Whereas CRP, fibrinogen and WBC are associated with the promotion of inflammation, IGF-1 has anti-inflammatory properties. It is therefore of note that aspects of social engagement were associated with reduced inflammation whilst loneliness was inversely related to a marker involved in the regulation of inflammation. This suggests that there could be different biological pathways involved in objective and subjective aspects of social connection. However, it is important to note that although the same pattern of these results for WBC and IGF-1 were found when analysing data that did not include the imputed data for wave 2, significance was not. So this finding remains to be explored further.

The major strength of our investigation was the use of fixed-effects regressions that account for time-varying covariates. Unlike previous studies, our analysis examined the relationship between changes in biomarkers and changes in social engagement. Many analyses in this area cannot account for early life, genetic or personality based factors that may influence social exposures and biomarker outcomes. Yet by using each participant as their own control, the influence of these time invariant factors was eliminated from our analysis. In addition, our independent analysis of a range of aspects of social connection give a detailed and holistic description of social engagement in old age. Many studies on this topic examine one type of social isolation and so have been unable to identify specific responses to different types of social exposures.

However, our results are observational so causality cannot be assumed, especially as unobserved time-varying confounders could still explain results. Our findings can also only be generalised to the target population of ELSA. Considering the reported cross-cultural differences in social activities in older age, more research is needed to investigate whether our findings can be generalised to other populations (Jylhä and Jokela, 1990). In addition, the WBC and IGF-1 results could be an artefact of the multiple imputation as values for these markers were entirely imputed at wave 2. However, as wave 2 data were missing due to financial limitations on the number of biomarkers that were tested rather than on selective participation, we can assume this was therefore missing at random, and a comprehensive list of variables were used to predict the values. Further, the findings for WBC are mirrored in findings for CRP and figrinogen, for which data were available for every wave. Additionally, we were limited in this study to analyses of neuro-immune markers that had been collected as part of ELSA. Future studies could explore whether the pattern of findings is corroborated when considering a broader panel of immune biomarkers. Finally, we adjusted for a range of chronic conditions in our analyses and excluded participants with high levels of CRP that could indicate an acute infection or serious acute illness, but we were unable to control for autoimmune conditions.

Overall, these results suggest that there could be different biological pathways involved in objective and subjective aspects of social connections, with social engagement and living with somebody longitudinally associated with lower levels of the pro-inflammatory markers CRP, fibrinogen and WBC, and lower levels of loneliness associated with higher levels of anti-inflammatory IGF-1. These results are significant given that inflammation in older age is associated with greater risk for various inflammatory-related diseases including cardiovascular disease, diabetes, arthritis and certain cancers (The Emerging Risk Factors Collaboration, 2012), whilst increased WBC has been linked to earlier morbidity and mortality (Asadollahi et al., 2010). This risk can be exacerbated if socially-isolated individuals additionally experience acute stressors, which have been shown to lead to higher inflammatory responses than amongst individuals who have strong social ties (Moieni et al., 2015, Steptoe et al., 2007). In considering how to enhance social engagement, this study focused on activities including socialising, community group membership and going to cultural events. These are all modifiable factors, especially given increases in social prescribing, which refers individuals to social activities (Kimberlee, 2016). Therefore, future studies could explore whether social interventions for older adults could help in the prevention of biological changes associated with the onset of mental and physical poor health.

## Declaration of Competing Interest

The authors declare that they have no known competing financial interests or personal relationships that could have appeared to influence the work reported in this paper.
