# Supplementary material for: Social engagement and loneliness are differentially associated with neuro-immune markers in older age: Time-varying associations from the English Longitudinal Study of Ageing
Source: Brain Behav Immun. 2019 Nov;82:224–9. doi: 10.1016/j.bbi.2019.08.189 (PMC6997881; doi:10.1016/j.bbi.2019.08.189)
Supplement: Supplementary data 1 [file mmc1.docx]

Supplementary Material

Supplementary Table 1: Exposures and outcomes across the three time points

|  |  | Mean | SD | % |
| --- | --- | --- | --- | --- |
| Exposures | |  |  |  |
| Social engagement | Wave 2 | 7.70616 | 1.950117 |  |
|  | Wave 4 | 7.996771 | 1.944459 |  |
|  | Wave 6 | 8.114624 | 1.929387 |  |
| Living with somebody | Wave 2 |  |  | 26.60% |
|  | Wave 4 |  |  | 20.80% |
|  | Wave 6 |  |  | 18.90% |
| Loneliness | Wave 2 | 7.508192 | 1.370192 |  |
|  | Wave 4 | 7.664868 | 1.376402 |  |
|  | Wave 6 | 7.789635 | 1.375144 |  |
| Outcomes | |  |  |  |
| CRP | Wave 2 | 1.140175 | 0.57808 |  |
|  | Wave 4 | 1.059643 | 0.582012 |  |
|  | Wave 6 | 0.996824 | 0.568997 |  |
| Fibrinogen | Wave 2 | 3.246227 | 0.706359 |  |
|  | Wave 4 | 3.249048 | 0.636444 |  |
|  | Wave 6 | 3.054264 | 0.622582 |  |
| White blood cells | Wave 2 | 6.382862 | 2.042776 |  |
|  | Wave 4 | 6.272724 | 2.025761 |  |
|  | Wave 6 | 6.231481 | 2.008396 |  |
| IGF-1 | Wave 2 | 15.49269 | 5.418428 |  |
|  | Wave 4 | 15.51412 | 5.487634 |  |
|  | Wave 6 | 15.59844 | 5.273626 |  |

Sensitivity analysis 1: Results from fixed-effects regression models showing time-varying associations between loneliness, engagement, living alone and neuro-immune markers, additionally adjusted for BMI

|  | **CRP (95% CI)** | | **Fibrinogen (95% CI)** | | **WBC (95% CI)** | | **IGF-1 (95% CI)** | |
| --- | --- | --- | --- | --- | --- | --- | --- | --- |
|  | Coef (95% CI) | p | Coef (95% CI) | p | Coef (95% CI) | p | Coef (95% CI) | p |
| Social engagement | -0.007 (-0.015 to 0.001) | .072 | **-0.012 (-0.021 to -0.003)** | **.007** | **-0.040 (-0.079 to -0.002)** | **.038** | -0.026 (-0.124 to 0.072) | .60 |
| Living with somebody | **-0.072 (-0.110 to -0.035)** | **<.001** | **-0.104 (-0.153 to -0.054)** | **<.001** | **-0.257 (-0.434 to -0.079)** | **.005** | 0.321 (-0.148 to 0.072) | .18 |
| Low levels of loneliness | -0.001 (-0.013 to 0.010) | .81 | 0.0002 (-0.012 to 0.012) | .98 | -0.012 (-0.058 to 0.034) | .61 | **0.133 (0.026 to 0.239)** | **.015** |

Number of observations: 26,340; number of individuals: 8,780; observations per group: 3. **Higher** **scores indicate greater social engagement, living with others and lower levels of loneliness.** Model accounted for all time-invariant factors, even if unobserved, and adjusted for time-varying demographic covariates (marital status, employment status, wealth), health-related factors (presence of a long standing illnesses, long term pain, alcohol consumption, smoking status, sedentary behaviours), depression and BMI.

Sensitivity analysis 2: Results from fixed-effects regression models showing time-varying associations between loneliness, engagement, living alone and neuro-immune markers, each exposure entered independently into models

|  | **CRP (95% CI)** | | **Fibrinogen (95% CI)** | | **WBC (95% CI)** | | **IGF-1 (95% CI)** | |
| --- | --- | --- | --- | --- | --- | --- | --- | --- |
|  | Coef (95% CI) | p | Coef (95% CI) | p | Coef (95% CI) | p | Coef (95% CI) | p |
| Social engagement | -0.007 (-0.015 to 0.001) | .087 | **-0.012 (-0.021 to -0.003)** | **.007** | **-0.041 (-0.079 to -0.004)** | **.032** | -0.017 (-0.115 to 0.082) | .74 |
| Living with somebody | **-0.060 (-0.099 to -0.022)** | **.002** | **-0.100 (-0.149 to -0.051)** | **<.001** | **-0.252 (-0.426 to -0.079)** | **.005** | 0.404 (-0.060 to 0.867) | .087 |
| Low levels of loneliness | -0.006 (-0.018 to 0.005) | .27 | -0.006 (-0.018 to 0.006) | .35 | -0.028 (-0.073 to 0.016) | .21 | **0.140 (0.032 to 0.248)** | **.011** |

Number of observations: 26,340; number of individuals: 8,780; observations per group: 3. **Higher** **scores indicate greater social engagement, living with others and lower levels of loneliness.** Model accounted for all time-invariant factors, even if unobserved, and adjusted for time-varying demographic covariates (marital status, employment status, wealth), health-related factors (presence of a long standing illnesses, long term pain, alcohol consumption, smoking status, sedentary behaviours), and depression.

Sensitivity analysis 3: Results from fixed-effects regression models showing time-varying associations between loneliness, engagement, living alone and neuro-immune markers, excluding individuals with an infection (n=10)

|  | **CRP (95% CI)** | | **Fibrinogen (95% CI)** | | **WBC (95% CI)** | | **IGF-1 (95% CI)** | |
| --- | --- | --- | --- | --- | --- | --- | --- | --- |
|  | Coef (95% CI) | p | Coef (95% CI) | p | Coef (95% CI) | p | Coef (95% CI) | p |
| Social engagement | -0.006 (-0.014 to 0.002) | .15 | **-0.012 (-0.021 to -0.003)** | **.012** | **-0.039 (-0.078 to -0.004)** | **.048** | -0.024 (-0.123 to 0.075) | .63 |
| Living with somebody | **-0.049 (-0.089 to -0.009)** | **.016** | **-0.093 (-0.144 to -0.041)** | **<.001** | **-0.217 (-0.399 to -0.035)** | **.02** | 0.326 (-0.153 to 0.806) | .18 |
| Low levels of loneliness | -0.004 (-0.016 to 0.008) | .49 | -0.001 (-0.014 to 0.011) | .85 | -0.011 (-0.058 to 0.036) | .64 | **0.136 (0.021 to 0.250)** | **.021** |

Number of observations: 24,760; number of individuals: 8,770; observations per group: 3. **Higher** **scores indicate greater social engagement, living with others and lower levels of loneliness.** Model accounted for all time-invariant factors, even if unobserved, and adjusted for time-varying demographic covariates (marital status, employment status, wealth), health-related factors (presence of a long standing illnesses, long term pain, alcohol consumption, smoking status, sedentary behaviours), and depression.

Sensitivity analysis 4: Results from fixed-effects regression models showing time-varying associations between loneliness, isolation, living alone and neuro-immune markers, weighted using survey weights

|  | **CRP (95% CI)** | | **Fibrinogen (95% CI)** | | **WBC (95% CI)** | | **IGF-1 (95% CI)** | |
| --- | --- | --- | --- | --- | --- | --- | --- | --- |
|  | Coef (95% CI) | p | Coef (95% CI) | p | Coef (95% CI) | p | Coef (95% CI) | p |
| Social engagement | -0.007 (-0.015 to 0.002) | .11 | **-0.012 (-0.021 to -0.003)** | **.011** | **-0.040 (-0.079 to -0.002)** | **.041** | -0.026 (-0.127 to 0.074) | .60 |
| Living with somebody | **-0.057 (-0.098 to -0.016)** | **.006** | **-0.095 (-0.147 to -0.042)** | **<.001** | **-0.239 (-0.417 to -0.062)** | **.009** | 0.349 (-0.132 to 0.831) | .16 |
| Low levels of loneliness | -0.004 (-0.016 to 0.008) | .52 | -0.001 (-0.014 to 0.012) | .87 | -0.016 (-0.063 to 0.031) | .51 | **0.131 (0.022 to 0.240)** | **.018** |

Number of observations: 26,340; number of individuals: 8,780; observations per group: 3. **Higher** **scores indicate greater social engagement, living with others and lower levels of loneliness.** Model accounted for all time-invariant factors, even if unobserved, and adjusted for time-varying demographic covariates (marital status, employment status, wealth), health-related factors (presence of a long standing illnesses, long term pain, alcohol consumption, smoking status, sedentary behaviours), and depression.

Sensitivity analysis 5: Results from fixed-effects regression models showing time-varying associations between loneliness, isolation, living alone and neuro-immune markers, excluding the imputed data for WBC & IGF-1 at wave 2

|  | **WBC-1 (95% CI)** | | **IGF-1 (95% CI)** | | |
| --- | --- | --- | --- | --- | --- |
|  | Coef (95% CI) | p | Coef (95% CI) | p |  |
| **Model 1: accounting for all time-invariant factors** | | |  |  |  |
| Social engagement | **-0.062 (-0.104 to -0.020)** | **.004** | 0.017 (-0.096 to 0.130) | .77 |  |
| Living with somebody | **-0.528 (-0.768 to -0.288)** | **<.001** | **0.823 (0.204 to 1.442)** | **.009** |  |
| Low levels of loneliness | -0.046 (-0.100 to 0.008) | .092 | **0.143 (0.010 to 0.275)** | **.035** |  |
| **Model 2: additionally adjusted for time-varying demographic factors** | | |  |  |  |
| Social engagement | **-0.049 (-0.092 to -0.006)** | **.027** | -0.017 (-0.133 to 0.099) | .78 |  |
| Living with somebody | **-0.380 (-0.629 to -0.131)** | **.003** | 0.401 (-0.222 to 1.024) | .21 |  |
| Low levels of loneliness | -0.032 (-0.086 to 0.022) | .24 | 0.107 (-0.026 to 0.240) | .11 |  |
| **Model 3: additionally for time-varying health-related factors** | | |  |  |  |
| Social engagement | -0.034 (-0.076 to 0.009) | .12 | -0.019 (-0.138 to 0.099) | .75 |  |
| Living with somebody | -0.234 (-0.483 to 0.015) | .066 | 0.337 (-0.285 to 0.959) | .29 |  |
| Low levels of loneliness | -0.017 (-0.070 to 0.036) | .52 | 0.101 (-0.033 to 0.234) | .14 |  |
| **Model 4: additionally for time-varying depression** | | |  |  |  |
| Social engagement | -0.033 (-0.076 to 0.009) | .13 | -0.019 (-0.138 to 0.099) | .75 |  |
| Living with somebody | -0.224 (-0.476 to 0.028) | .081 | 0.336 (-0.287 to 0.959) | .29 |  |
| Low levels of loneliness | -0.014 (-0.068 to 0.039) | .60 | 0.100 (-0.036 to 0.237) | .15 |  |

Number of observations: 17,560; number of individuals: 8,780; observations per group: 2. **Higher** **scores indicate greater social engagement, living with others and lower levels of loneliness.** Model 1 accounted for all time-invariant factors, even if unobserved. Model 2 adjusted for time-varying demographic covariates (marital status, employment status, wealth). Model 3 additionally adjusted for time-varying health-related factors (presence of a long standing illnesses, long term pain, alcohol consumption, smoking status, sedentary behaviours). Model 4 additionally adjusted for depression.
